# Editorial: Apoptosis and Senescence in Vertebrate Development

**DOI:** 10.3389/fcell.2021.834517

**Published:** 2022-01-05

**Authors:** Wolfgang Knabe, Stefan Washausen

**Affiliations:** Prosektur Anatomie, Westfälische Wilhelms-Universität Münster, Münster, Germany

**Keywords:** apoptosis, programmed cell death, cellular senescence, autophagy, macrophages, embryonic development, regeneration, vertebrates

## Introduction

In the history of developmental biology, apoptosis and senescence will foreseeably share at least four characteristics. Firstly, both have made their way into this field after detours. Thus, dead cells were admittedly discovered early within embryos of various vertebrate classes, well before the term apoptosis came into use (Kerr et al., 1972). Conceptually, however, ontogenetic cell death events were accepted and interpreted hesitantly, despite their meticulous temporal and spatial descriptions (Rabl, 1898-1899; Rabl, 1899-1900; Jokl, 1918, 1920; Ernst, 1926; Peter, 1936; Glücksmann, 1951). Correspondingly, the term senescence did not even originate from developmental biology at all, but rather from ageing research (Hayflick and Moorhead, 1961), and it was not until about 50 years later that contributions of permanent cell cycle arrests to embryonic development received adequate recognition (Muñoz-Espín et al., 2013; Storer et al., 2013). A second common feature of apoptosis and senescence is that both are part of their own wide spectrum of different forms. Thus, we are far from confident today that cell death events found during normal and/or disturbed development generally belong to the category “apoptosis”. Rather, it could alternatively or additionally be necroptosis, parthanatos, entotic cell death, lysosome-dependent cell death, autophagy-dependent cell death, or linker cell-type death, to name just a few candidates (Yuan and Kroemer, 2010; Kutscher and Shaham, 2017; Galluzzi et al., 2018; Voss and Strasser, 2020). Likewise, differently mediated types of senescence are induced by aging, oncogenes, irradiation, chemotherapy, tissue damage, induced pluripotent stem cell reprogramming or developmental cues (Muñoz-Espín and Serrano, 2014; Herranz and Gil, 2018; da Silva-Álvarez et al., 2019; Rhinn et al., 2019; Varela-Nieto et al., 2019). Considering these diversities of forms, a third similarity between apoptosis and senescence can be compellingly deduced. Indeed, a whole arsenal of detection methods must be employed to identify apoptotic or senescent cells, respectively (Vanden Berghe et al., 2013; Majtnerová and Roušar, 2018; González-Gualda et al., 2021). Finally, a fourth common feature consists in the fact that we still know little about the functions of apoptosis and senescence in many developmental contexts. Especially, presumed regulatory and functional interrelationships between these two processes are still largely obscure. This Research Topic attempts to address the above described situation as follows. Our primary goal was to present new evidence for the occurrence and functions of programmed cell death/apoptosis and/or cellular senescence during normal vertebrate development. In order to promote interdisciplinary bridging, contributions to the fields “postnatal development”, “*in-vitro* development”, “aging”, “disease”, and “regeneration” were additionally included. Other papers critically investigate the significance of methods used to detect developmental senescence, or “canonical” and “non-canonical” roles of components of the apoptosis machinery.

## Summary of the Articles

This Research Topic includes 20 articles written by 118 authors and reviewed by 41 referees, who in their entirety came from 25 different countries and six continents. The following section presents the focus and key findings of each article.

We open our Research Topic with a review on senescence and apoptosis as the architects of mammalian development (Wanner et al.). This overview starts by addressing zygotes and blastocysts, which are later revisited under *in vitro* conditions (Ramos-Ibeas et al.; Yu et al.). In the opposite direction, the chronological spectrum even covers transitions to ageing processes. Further main topics are epigenetic modifications and the ambivalence of several tumor suppressors [p53, Retinoblastoma (Rb), Inhibitor of Growth (ING)] which “play antagonistic roles by increasing fitness and decreasing mortality early in life but contribute to deleterious effects and pathologies later in life” or, in short, which reveal “antagonistic pleiotropy” (Wanner et al.).

Subsequently, de Mera-Rodríguez et al. examine to which extent senescence-associated ß-galactosidase and the cyclin-dependent kinase (CDK) inhibitor p21 are reliable *in vivo* markers of cellular senescence. The authors focus on retinal and limb development, but also outline other organ systems. Possible interrelationships between apoptosis and senescence are additionally considered. Given that ß-galactosidase activity overlaps with areas of cell death and, like p21, is also present in newly differentiated retinal neurons, both “senescence markers” may be less specific than previously thought. Ultimately, methodological concerns highlighted here underscore the recent postulate that proper detection of senescence must be based on experiments using at least three independent markers (González-Gualda et al., 2021).

We continue with a series of eight papers dealing with the nervous system, sense organs and craniofacial development. Firstly, Nie et al. revisit previously published work on the contributions of sumoylation processes to optic lens development (Yan et al., 2010; Gong et al., 2014). The question now is whether sumoylation is significant also during oxidative stress-induced cataractogenesis. In fact, the authors show that the E3 ligase PIAS1 (protein inhibitor of activated STAT-1) regulates p53 sumoylation to control stress-induced apoptosis of lens epithelial cells through the pro-apoptotic regulator Bax.

In the second place, Domínguez-Bautista et al. report on programmed cell senescence in the spinal cord and notochord of mouse embryos. They provide new evidence that senescent cells express different markers in different developmental contexts, in this case either the CDK inhibitors p16 (spinal motoneurons) or p21 (endothelial cells, notochord). Furthermore, it is demonstrated that senescent cells in the floor plate signaling centre (Muñoz-Espín et al., 2013) are joined by quiescent cells. Given that treatment with senolytics led to a reduction in the number of spinal motoneurons, Domínguez-Bautista et al. conclude that “programmed cell senescence cooperates with apoptosis to adjust the number of motoneurons”.

The third paper in this series examines the anti-apoptotic role of the Bcl2 protein Mcl-1 (Myeloid cell leukemia-1) in the embryonic spinal cord, brainstem and forebrain (Flemmer et al.). Using the *Nestin-Cre Mcl-1* conditional knockout (*Mcl-1* CKO) mouse line, the authors detected apoptotic waves which, in the spinal cord, proceeded ventrodorsally and rostrocaudally from embryonic day 9 onwards. Consequently, Mcl-1 does not contribute to neural tube formation, but to the differentiation of neuronal precursor cells (NPC) into immature neurons. Breeding *Mcl-1* CKO mice with *Bax* null mice further demonstrated “that Mcl-1 promotes NPC survival primarily by inhibiting the activation of Bax, but that Bax is not the sole pro-apoptotic target of Mcl-1 during embryonic neurogenesis” (Flemmer et al.).

Following this, Nguyen et al. address the fact that caspases do not only perform “canonical” functions in the context of apoptosis, but also “non-canonical” functions in axon guidance, axon pruning, synapse maturation, and synaptic transmission. Furthermore, this review takes into account the signaling pathways that select between or execute apoptotic or non-apoptotic responses. Some of these signaling pathways are additionally explored in original papers belonging to this Research Topic (e.g.: Mcl-1: Flemmer et al.; Bax: Nie et al.; WNT, BMP, FGF: Díaz-Hernández et al.).

The fifth paper raises the question of how ethanol-induced apoptosis can be prevented in human neural crest cells (Li et al.). It thus continues earlier work documenting the occurrence of increased apoptosis in the nervous system, head and limbs of ethanol-exposed embryonic mice (Kotch et al., 1992, Kotch and Sulik, 1992a, Kotch and Sulik, 1992b; Kotch et al., 1995; Dunty et al., 2001, 2002). Here, an example is provided of how anti-apoptotic genes can be epigenetically suppressed (for a review, see Wanner et al.). Specifically, the vegetable-derived isothiocyanate sulforaphane “protects against ethanol-induced apoptosis (…) through diminishing ethanol-induced hypermethylation at the promoters of the genes encoding the inhibitor of apoptosis proteins” (Li et al.).

Next, Compagnucci et al. set out how apoptosis affects normal and abnormal craniofacial development. Continuity with previously featured teratological studies is obvious, as approximately one third of all human congenital malformations are manifested in the head and face (Twigg and Wilkie, 2015). The authors focus on epithelial-epithelial apposition, intraepithelial morphogenesis, epithelial compartmentalization and metameric organization of the cranial neural crest, exemplified by the jaw modules, the lambdoidal junction, the pharyngeal arch 1 hinge, and the upper jaw, respectively. Overall, developmental apoptosis appears to work less with the “sledgehammer” and much more as “subtle sculptor”. Entirely in line with our rationale for launching this Research Topic, another conclusion is that “a comprehensive exposition of the normal, predictable spatiotemporal ontogeny of apoptosis explicitly in the craniofacial tissues of vertebrate embryos has yet to materialize” (Compagnucci et al.).

We proceed with early tooth development, which has emerged as an attractive model in mesenchymal stem cell research (Yu et al., 2015; Sharpe, 2016). Here, Kim et al. demonstrate that hypoxia-responsive oxygen nanobubbles prevent hypoxia-induced pathological increases of apoptosis in the cap and bell stages of mouse tooth germs. It can therefore be assumed that hypoxia-responsive oxygen nanobubbles will be suitable for regenerative approaches in the dental field. Whether, in tooth germs, hypoxia-inducible factor-1 (HIF-1; Kietzmann et al., 2001) may also trigger senescence, possibly via p53, remains to be investigated.

The subsequent paper by Maeda starts by reviewing the hard tissues of adult teeth which all display unique structural, molecular and physiological senescence patterns. From here, Maeda builds bridges to cellular senescence of dental pulp (stem) cells including tooth germs, but also to the topic of regeneration. Undoubtedly, “dental pulp stem cells (…) have attracted considerable attention as promising cells for regenerative endodontics (…) and systemic regenerative medicine” (Maeda).

The following group of three articles is dedicated to trunk and limb development. We begin with a study on the teratogenic effects of caffeine in chicken embryos (Wu et al.). Caffeine exposure interferes with the expression of genes modulating apoptosis, proliferation, and differentiation of myogenic progenitors in differentiating somites as well as in the chest wall. Resembling the case of ethanol-treated human neural crest cells (Li et al.), increased apoptosis affected caffeine-treated myogenic progenitors. Unexpectedly, however, significantly increased proliferation rates were additionally recorded under the influence of caffeine. All pathological changes were suppressible by the administration of retinoic acid antagonists.

The second article illustrates that at least two different mechanisms are involved in the degradation of superfluous cells during digit formation (Montero et al.). Apoptosis seems to dominate the onset of interdigital tissue remodeling, but is later coincident with “destructive developmental cell senescence”, which, however, is difficult to distinguish from other lysosomal pathway-mediated processes. A second type of (true?) senescence observed during digit formation shows no association with apoptosis, but is most likely related to tendon differentiation (“constructive developmental senescence”). Overall, the authors are certainly right to warn against overly rapid classification attempts in cases of cellular degradation pathway confluence.

The article closing the limb topic deals with the “anterior necrotic zone” (ANZ) which, in chicken embryos, promotes digit number reduction (Díaz-Hernández et al.). The authors show that cell death in the ANZ can be triggered either by inhibition of FGF or WNT signaling, or by activation of BMP signaling. Surprisingly, however, FGF signaling induces the expression of the pro-apoptotic genes *Dickkopf* (*Dkk*) and *Bmp4*. Furthermore, FGF8 in the apical ectodermal ridge and BMP4 in the anterior border of the limb are inhibited by BMP4. If, on the other hand, BMP4 is blocked by NOGGIN, *Dkk* expression in the ANZ gets reduced. Double treatment experiments support the hypothesis that during BMP4 activation, FGF signaling may represent the final step in the molecular cascade leading to early cell death in the ANZ (Díaz-Hernández et al.).

The following original paper highlights the importance of the ribosome biogenesis factor LTV1 for digestive organ development and hematopoiesis in zebrafish (Zhang et al.). In our thematic context, ribosomopathies are attractive, among other aspects, because they can trigger apoptosis or senescence via p53 (Fumagalli and Thomas, 2011; Turi et al., 2019; Da Costa et al., 2020). In *ltv1* knockouts, however, defects of exocrine pancreas as well as hematopoietic stem and progenitor cells were not associated with increased apoptosis, but with decreased growth rates. Also, a possible dependence on p53 could not be confirmed.

Three other papers explore senescence and/or apoptosis at particularly early developmental stages. At the outset, Palomino et al. take up the earlier observation, relevant not least to global fishing industry, that captive yellow-tail kingfish (*Seriola lalandi*) show lower survival rates due to the failure of their buoyancy system. Now, the authors report that animals living in captivity (stages 2/4C, morula, blastula, gastrula, and 24 hpf) exhibit massively increased apoptosis rates. In line with this, changes in the expression levels of cell death-associated genes are demonstrated (*bax*, *casp9*, *casp3*, *casp8*, Fas).

Next is a review on the occurrence of senescence and apoptosis during *in vitro* bovine embryo development (Ramos-Ibeas et al.). In pre-implantational embryos, protective senescence initially gains regulatory significance. Later during morula and blastula stages, apoptosis is in the foreground, whereby “embryonic quality is normally associated with lower apoptotic rates”. Main parts of this work provide practical guidance on how to assess embryo quality using structural criteria and molecular markers for measuring senescence and apoptosis.

Correspondingly, Yu et al. address the question on how to predict bovine blastocyst quality. 42% of the blastocysts examined show cellular fragments in the perivitelline space before transfer, and 37% of these fragments are TUNEL-negative, i.e. non-apoptotic. Furthermore, neither correlative nor causal relationships were found between the occurrence of perivitelline fragments and blastocyst quality. The authors therefore conclude “that apoptosis and fragmentation are methods to remove abnormal cells from the embryo, and thereby protect the developmental competence of the embryo”.

As with the retina (de Mera-Rodríguez et al.), we extend the scope of our Research Topic one last time to the postnatal developmental period, this time using the skin as an example. Specifically, Chou et al. intend to establish a mouse model that recapitulates key pathological features of human diseases associated with dysfunctions of the tumor suppressor gene *Wwox*. They show that *Wwox* deficiency leads to decreases in the proliferation and differentiation of keratinocytes as well as to increased apoptosis rates in *Wwox* ^−/−^ mouse epidermis, primary keratinocyte cultures and *Wwox* knockdown human HaCaT cells. Most probably, *Wwox* deficiency is mediated by the downregulation of prosurvival MEK/ERK signaling.

The two concluding articles of this Research Topic focus on possible links between apoptosis, senescence, development and regeneration. Firstly, Li et al. present a systematic summary of the different types of apoptotic extracellular vesicles (ApoEVs) which contain nucleic acids, protein lipids, mitochondria, and plasma membrane, among others. Possible developmental and/or regenerative functions of ApoEVs include the apoptosis-induced activation of compensatory proliferation and/or differentiation processes as well as the elimination of senescent cells. Interestingly, senescent cells, owing to their senescence-associated secretory phenotype (SASP), display fascinating parallels to ApoEVs by releasing cytokines, growth factors or DNA fragments into their environment.

Molecular similarities between apoptosis-induced regenerative and developmental processes are also explored in the final review prepared by Guerin et al. Typical subjects of discussion are functions of BMPs in the limbs or those of Jun-N terminal kinase (JNK) in the brain. Furthermore, the importance of apoptosis for cell signaling and patterning mechanisms is being investigated. Overall, the authors conclude that, despite numerous commonalities, apoptosis-induced regeneration by no means simply recapitulates apoptosis-induced developmental mechanisms.

## Perspectives

Taken together, the results of this Research Topic first of all show that numerous previously undescribed cases of ontogenetic cell death still await discovery. Furthermore, there is increasing evidence that developmental events may not be propelled by one type of cell death alone, but that several different types of cell death may be involved, either simultaneously or successively. We conclude from this that, even in developmental contexts where, for example, apoptosis safely fulfils specific functions, we should nevertheless explore the remaining spectrum of cell death forms. This need is all the more acute in cases where the functions of ontogenetic cell death are still unclear, or where experimental suppression of programmed cell death entails inconclusive results. Especially in the latter instance, switching between several types of cell death might be part of the embryo’s “survival repertoire”. Understanding such possible cell death transitions might also help to better appreciate similarities and differences between developmental and regenerative processes.

With the discovery of programmed senescence, the situation initially became even more complicated for developmental biologists interested in programmed cell death. That is why it is all the more imperative now to meticulously document examples of the complementary, simultaneous and/or successive occurrence of both processes in well-defined developmental contexts. We are convinced that without such systematic investigations it will hardly be possible to gain experimentally validated insights into the functional interactions of programmed cell death and senescence. To achieve these objectives, as in the case of programmed cell death, it will undoubtedly be necessary to solve numerous methodological problems that currently hamper the reliable diagnosis of senescent cells. Conversely, our ever-increasing knowledge about the regulatory potential of secretory active senescent cells will certainly inspire developmental biologists to take a fresh look at the developmentally relevant functions of ApoEvs as well as at the non-canonical functions of components of the apoptosis machinery.

Finally, we are left with the pleasant duty to express our sincere thanks to all those who helped to realize this Research Topic. We were particularly impressed by the constant willingness of both our authors and reviewers to contribute ambitiously to the optimization of submitted manuscripts through precise, critical and self-critical comments, questions and responses. Unwanted by all, the Corona pandemic has also left its marks on our project, as much through months of laboratory closures as through severe diseases affecting families and friends. It was therefore all the more encouraging to learn how much trust and tolerance still prevail in the scientific community. Especially the latter experience would never have become reality without the always excellent cooperation with the Frontiers Editorial Team.
